# Interferon-γ and its pathway-associated gene expression in the vaginal tissue of premenopausal females with pelvic organ prolapse

**DOI:** 10.3892/etm.2014.1868

**Published:** 2014-07-29

**Authors:** BING ZHAO, JIANGUO YAN, HUIYAN WU, YALI ZHOU, DONGMEI XU, MENGCAI HU, SHIHONG CUI

**Affiliations:** 1 The Third Affiliated Hospital of Zhengzhou University, Zhengzhou, Henan 450052, P.R. China; 2 Department of Anatomy, Basic Medical College, Xinxiang Medical University, Xinxiang, Henan 453003, P.R. China

**Keywords:** pelvic organ prolapse, interferon-γ, gene expression

## Abstract

Interferon (IFN)-γ is a potent proinflammatory molecule. However, few studies have investigated the expression levels of IFN-γ during pelvic organ prolapse (POP). In the present study, the expression levels and tissue localization of IFN-γ and its pathway-associated genes were detected in the vaginal walls of premenopausal females with POP and asymptomatic controls using quantitative polymerase chain reaction and immunohistochemistry. When compared with the matched controls, an 8.6-fold increase in IFN-γ, 3.8-fold increase in IFN-γ receptor (IFNGR)1, 2.6-fold increase in IFNGR2, 3.4-fold increase in signal transducer and activator of transcription-1, 2.2-fold increase in janus kinase-1 and 5.1-fold increase in nuclear factor (NF)-κB mRNA expression levels were observed in the females with premenopausal POP. In all the females with POP, higher mRNA expression levels of IFN-γ and its receptors were observed when compared with the controls. Expression levels of all the proteins were detected by immunohistochemistry, and the results demonstrated higher staining for IFN-γ, IFNGRs and pathway-associated genes in females with POP. Therefore, the results indicated that IFN-γ may be used as an inflammatory marker for POP development, and is associated with NF-κB.

## Introduction

Pelvic organ prolapse (POP), a hernia in the endopelvic fascia (1), is associated with urinary incontinence and defecation dysfunction, leading to an impaired quality of life for the affected individual (2). Epidemiological studies have revealed that numerous risk factors, including vaginal delivery, senescence, obesity and pelvic surgery, contribute to the development of the condition (3–5).

The pelvic organs are located in the pelvic floor, which consists of connective tissue in a network of tough extracellular matrix (ECM) protein fibers. To date, studies have primarily focused on the molecular and biochemical changes in POP, including ECM proteins (collagen, elastin and fibulin) (6,7) and enzymes (5). The results have indicated that the genes associated with the regulation of collagen fiber assembly and impairment in elastic fibers play an important role in the development of POP. Brizzolara *et al* (2) observed that several genes involved in immunity and defense, including interleukin (IL)-6, Toll-like receptors and interferon (IFN)-γ receptors (IFNGRs), were upregulated, indicating that these genes enriched for ‘immunity and defense’ contribute to POP.

IFNs were the first cytokines to be identified; thus, have provided the fundamental base from which the understanding of the functions, pathways, evolution and structure of other class II cytokines and their receptors began. IFNs were also the first cytokines to be used therapeutically (8). Based on their receptor specificity and sequence homology, IFNs are classified into type I, II and III. Type I IFNs consist of seven classes, including IFN-α, -β, -ɛ, -κ, -σ, -ω and -τ, while type II IFNs consist of IFN-γ only (8). IFN-γ is an important cytokine in the host defense against infection by viral and microbial pathogens. Following the binding of IFN-γ to its heterodimeric cell surface receptors, IFNGR1 and IFNGR2, the janus kinase (JAK)-signal transducer and activator of transcription (STAT) signaling pathway is activated. This activation is regulated by IFN regulatory factors (IRFs) and nuclear factor (NF)-κB. IFN-γ has been demonstrated to be involved in inflammatory disease progression, for example in rheumatoid arthritis (9). Type III IFNs include IFN-λ1, -λ2 and -λ3, which are also named as IL-29, IL-28A and IL-28B, respectively.

A previous study demonstrated that severe inflammation occurs during POP development (2). Thus, IFN-γ may be an optional inflammatory marker during the development of POP. The expression levels of IFN-γ may be associated with the levels of its pathway-related genes. Thus, in the present study, the gene expression levels and tissue localization of IFN-γ and its pathway-associated genes, including IFNGR1, IFNGR2, JAK1 and STAT1, were examined in the vaginal tissue of females with POP.

## Materials and methods

### Patient selection and tissue collection

Premenopausal females (n=12) undergoing surgery for POP were selected for involvement in the study according to their POP-quantification (POP-Q) staging system result, which was required to be stage II or greater (10). Females with a POP-Q score of stage 0 served as the controls (n=5). None of the participants in the POP or control groups suffered from stress urinary incontinence. All patient cases and controls were of Mongolian origin. The local Ethics Committee of the Third Affiliated Hospital of Zhengzhou University (Zhengzhou, China) approved the experimental procedures used in the study, and all female patients included provided informed consent.

During surgery, following the removal of the uterus ~1 cm^2^ was obtained by sharp dissection down to the avascular space of loose areolar tissue of the vagina using medical scissors for all the POP cases and controls. Specimens used for the quantitative polymerase chain reaction (qPCR) analysis were immediately placed in TRIzol reagent (Invitrogen Life Technologies, Carlsbad, CA, USA) and stored at −70°C. For immunohistochemical analysis, the specimens were fixed in 4% formaldehyde for 48 h.

### qPCR

Total RNA was extracted by TRIzol reagent and treated with 2.5 μl DNase I (Qiagen, Hilden, Germany). The RNA concentration was determined by measuring the optical density (OD)_260_/OD_280_ ratio until a value of 1.7 was obtained (Eppendorf AG, Hamburg, Germany), and by electrophoresis on 1.5% agarose gels. The RNA was reverse transcribed into cDNA using SuperScript^™^ RNase H-Reverse Transcriptase (Invitrogen Life Technologies), according to the manufacturer’s instructions.

qPCR was conducted on an Applied Biosystems 7500 Real-Time PCR system (Invitrogen Life Technologies) using Applied Biosystems SYBR^®^ Green Master mix (Invitrogen Life Technologies). All measurements were analyzed in triplicate on 96-well optical PCR-plates. Human ribosomal 18S rRNA (GenBank accession no. 4310893) was used as an internal standard. The qPCR protocol involved 40 cycles of denaturation-annealing. Following qPCR, a dissociation curve was constructed by increasing the temperature from 65 to 95°C in order to verify the specificity of the PCR products. The threshold cycles (Cts) were recorded and the relative quantitation (^ΔΔ^Ct method) was calculated to compare gene expression (11). The mRNA expression levels for POP were calculated as fold changes (FCs) relative to the control mRNA expression levels.

### Immunohistochemistry

Samples were sectioned at a thickness of 5 μm and mounted on glass. Antigen retrieval was performed by treatment with 0.125% trypsin, followed by blocking with 5% normal bovine serum and overnight incubation with the primary antibodies at 4°C; IFN-γ antibody (Santa Cruz Biotechnology Inc., Santa Cruz, CA, USA ), IFNGR1 antibody (Abcam Inc, London, UK), IFNGR2 antibody (Santa Cruz Biotechnology Inc.), JAK1 antibody (Santa Cruz Biotechnology Inc.), STAT1 antibody (Santa Cruz Biotechnology Inc.) and NF κB antibody (Cell Science, Sydney, Australia). This was followed by incubation with the appropriate anti-rabbit secondary antibodies (Wuhan Boster, Wuhan, China). For the negative control group, nonspecific rabbit IgG was used at the same concentration as the primary antibody (Santa Cruz Biotechnology Inc). The sections were counterstained with 10% Mayer’s hematoxylin, mounted and observed with a Leica microscope (Leica Microsystems, Ontario, Canada).

### Statistical analysis

Data were analyzed by one-way analysis of variance using the SAS 9.0 statistical software (SAS Institute, Inc., Cary NC, USA). P<0.05 was considered to indicate a statistically significant difference. Experimental error was reported as the standard error of the mean.

## Results

### Patient sample

Vaginal tissue samples were obtained from a total of 17 patients, of which 12 were patients with POP and 5 were control patients with vaginal myomas or other vaginal diseases. The patients were matched for age (50.6 vs. 47.5 years, respectively) and body mass index (25 vs. 26.8 kg/m^2^, respectively). The statistically significant differences were observed between the two groups in mean parity (3.8 vs. 1.9, respectively, P=0.001).

### Gene expression

**Figure 1:**
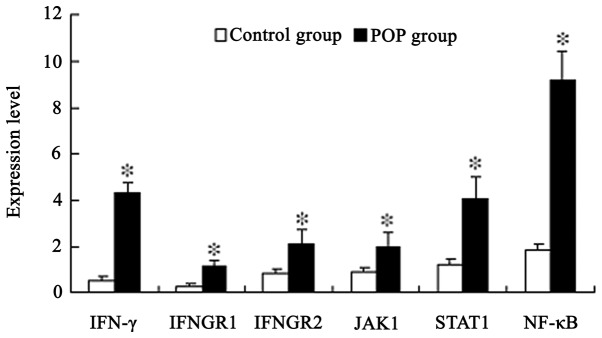
Quantitative polymerase chain reaction analysis of the expression levels of IFN-γ, IFNGR1, IFNGR2, JAK1, STAT1 and NF-κB in the control (n=10) and POP groups (n=12). Results are presented as the mean ± standard error of the mean. ^*^P<0.05, vs. control group. IFN, interferon; IFNGR, IFN-γ receptor; JAK, janus kinase; STAT, signal transducer and activator of transcription; NF, nuclear factor; POP, pelvic organ prolapse.

Gene expression levels were analyzed by qPCR. Gene expression levels of IFN-γ (qPCR FC, 8.6), IFNGR1 (qPCR FC, 3.8), IFNGR2 (qPCR FC, 2.6), JAK1 (qPCR FC, 2.2), STAT1 (qPCR FC, 3.4) and NF-κB (qPCR FC, 5.1) were significantly increased in patients with POP when compared with the control patients (P=0.001, 0.005, 0.04, 0.002, 0.001 and 0.007, respectively; Fig. 1). All the primers used in the analysis are listed in Table I.

### Immunohistochemistry

**Figure 2:**
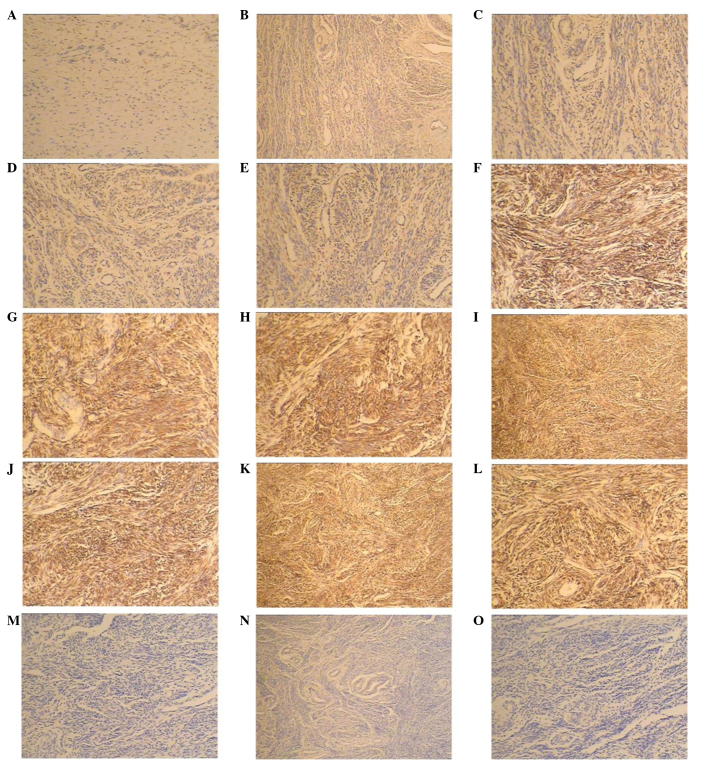
Immunohistochemical staining of the pelvic floor extracellular matrix showing genes in the control group: (A) Interferon (IFN)-γ, (B) IFN-γ receptor (IFNGR)1, (C) IFNGR2, (D) janus kinase (JAK)1, (E) signal transducer and activator of transcription (STAT)1 and (F) nuclear factor (NF)-κB; and genes in the pelvic organ prolapse group: (G) IFN-γ, (H) IFNGR1, (I) IFNGR2, (J) JAK1, (K) STAT1 and (L) NF-κB. (M-O) Immunohistochemical staining in the negative control group. The three images of the negative control demonstrate nonspecific rabbit IgG used at the same concentration as the primary antibody.

Levels of IFN-γ and its pathway-associated genes (IFNGR1, IFNGR2, JAK1, STAT1 and NF-κB) were detected by immunohistochemical analysis using specifically targeted antibodies. Immunohistochemical staining revealed that all the proteins were expressed in the ECM. Compared with the control group, the protein expression levels were upregulated in the POP group (Fig. 2).

## Discussion

Numerous genes, including actin, myosin, ECM-associated genes and transcription factors, have been studied and shown to be associated with POP (11–13). However, there have been a limited number of studies investigating the role of immune-associated genes in POP. In the present study, the mRNA and protein expression levels of IFN-γ, IFNGR1, IFNGR2 and their pathway-associated genes, JAK1, STAT1 and NF-κB, were demonstrated to increase in the ECM of the vaginal tissue of patients with POP, as compared with the control group patients.

The JAK-STAT signaling pathway is a general pathway involved in IFN activation. Initially, IFNs bind to their specific receptors: IFN-α receptor 1 (IFNAR1) and IFNAR2 for type I IFNs; IFNGR1 and IFNGR2 for IFN-γ; and IFN-λ receptor 1 and IL-10 receptor 2 for type III IFNs. Once the receptor complexes are fully assembled, the JAK-STAT signaling pathway is activated. Differences exist with regard to the interactions between STAT members and the different types of IFNs; for example, STAT1 and STAT2 interact with type I and type III IFNs, while STAT1 interacts with IFN-γ (14). In addition to the JAK-STAT signaling pathway, other pathways, including the NF-κB and mitogen-activated protein kinase (MAPK) signaling pathways, mediate the expression levels of IFNs (8).

The results of the present study demonstrated significantly increased levels of IFN-γ, IFNGR1 and IFNGR2 in the ECM of females with POP, which was in concordance with the observations of the study by Brizzolara *et al* (2). Combined with the results of this previous study, a hypothesis was proposed that the increase in the expression levels of IFN-γ and its receptors in females with POP is a result of the ECM NF-κB cascade. Conformational changes of the ECM in females with POP have been shown to result in the reorganization of the cellular cytoskeleton (15) and the activation of the MAPK (16) and NF-κB signaling pathways (17). To confirm this, the present study investigated the expression levels of NF-κB in the ECM using qPCR. The results clearly demonstrated that NF-κB expression was upregulated in patients with POP (FC, 5.1). These results were also supported by extensive analysis of the promoters of IFN-γ and its receptors, which are controlled almost entirely by IRFs and NF-κB.

The ECM component of connective tissue provides structural integrity and a three-dimensional scaffold. During POP etiopathology, inflammation was observed in the ECM. Although no inflammatory cells were observed, microarray studies have revealed that the expression levels of a number of inflammation-associated genes are elevated, indicating that the immune system plays a role in the etiopathology of POP and that there are alternative functions of these inflammatory processes (2).

IFN-γ, a T helper-1 cytokine, is not only a key mediator of antiviral defense, but also a mediator of inflammation. The cytokine has been shown to induce chemokines, including chemokine (C-X-C motif) ligand (CXCL)9 and CXCL10, which are associated with inflammation and disease progression (18). Using microarray techniques, Brizzolara *et al* (2) demonstrated that numerous immune-associated genes, including CXCL2 and CXC receptor 4, were strongly induced in POP. However, whether IFN-γ also plays a regulatory role in POP should be determined in future studies. In conclusion, the present study confirmed that IFN-γ and associated pathway genes were upregulated in patients with POP, and that this phenomenon was associated with NF-κB.

## Figures and Tables

**Table I:** Primer sequences.

Gene name	Sequence	Product size (bp)	Annealing temperature (°C)
IFN-γ	Forward 5′-GCAGGTCATTCAGATGTAGCGG-3′ Reverse 5′-GGCGACAGTTCAGCCATCACTT-3′	317	64
IFNGR1	Forward 5′-TATGTGAGAATGAACGGAAGTGA-3′ Reverse 5′-GATGAATACCAGGCTAAGCACTA-3′	276	59
IFNGR2	Forward 5′-AACATCTTTAGAGTCGGGCATTT-3′ Reverse 5′-TCTATCTGTAATGGGATGCATGG-3′	212	60
JAK1	Forward 5′-CCAGAACTGCCCAAGGACATCA-3′ Reverse 5′-ACGCTGCTGTCACAAATGGTCT-3′	158	63
STAT1	Forward 5′-GAGTGGAAGCGGAGACAGCAGA-3′ Reverse 5′-AGACTGAAGGTGCGGTCCCATA-3′	212	63
NF-κB	Forward 5′-ACCAAGGAGATGGACCTCAGCG-3′ Reverse 5′-CCTTCCCAGACTCCACCATTTT-3′	278	63
18S RNA	Forward 5′-GTCTTCACCACCATGGAGAAGGCT-3′ Reverse 5′-CATGCCAGCGAGCTTCCCGTTCA-3′	393	64

IFN, interferon; IFNGR, IFN-γ receptor; JAK, janus kinase; STAT, signal transducer and activator of transcription; NF, nuclear factor.
